# Genome-wide analysis of the CDPK gene family and their important roles response to cold stress in white clover

**DOI:** 10.1080/15592324.2023.2213924

**Published:** 2023-05-18

**Authors:** Manman Li, Xiuhua Chen, Wangqi Huang, Yanan Li, Qian Liu, Wei Yan, Changhong Guo, Yongjun Shu

**Affiliations:** a Key Laboratory of Molecular Cytogenetics and Genetic Breeding of Heilongjiang Province, College of Life Science and Technology, Harbin Normal University, Harbin, Heilongjiang, China; b International Agriculture Research Institute, Yunnan Academy of Agricultural Sciences, Kunming, Yunnan, China; c Flower Research Institute, Yunnan Academy of Agricultural Sciences, Kunming, Yunnan, China; d Institute of Tropical and Subtropical Cash Crops, Yunnan Academy of Agricultural Sciences, Baoshan, Yunnan, China

**Keywords:** White clover, CDPK, Genetic regulation network, Cold stress, RNA-seq

## Abstract

Calcium-dependent protein kinases (CDPKs) are an important class of calcium-sensitive response proteins that play an important regulatory role in response to abiotic stresses. To date, little is known about the CDPK genes in white clover. White clover is a high-quality forage grass with high protein content, but it is susceptible to cold stress. Therefore, we performed a genome-wide analysis of the CDPK gene family in white clover and identified 50 members of the CDPK genes. Phylogenetic analysis using CDPKs from the model plant Arabidopsis divided the TrCDPK genes into four groups based on their sequence similarities. Motif analysis showed that TrCDPKs within the same group had similar motif compositions. Gene duplication analysis revealed the evolution and expansion of TrCDPK genes in white clover. Meanwhile, a genetic regulatory network (GRN) containing TrCDPK genes was reconstructed, and gene ontology (GO) annotation analysis of these functional genes showed that they contribute to signal transduction, cellular response to stimuli, and biological regulation, all of which are important processes in response to abiotic stresses. To determine the function of TrCDPK genes, we analyzed the RNA-seq dataset and found that most TrCDPK genes were highly up-regulated under cold stress, particularly in the early stages of cold stress. These results were validated by qRT-PCR experiments, implying that TrCDPK genes are involved in various gene regulatory pathways in response to cold stress. Our study may help to further investigate the function of TrCDPK genes and their role in response to cold stress, which is important for understanding the molecular mechanisms of cold tolerance in white clover and improving its cold tolerance.

## Introduction

Throughout the life cycle of plants, their growth and development are often influenced by a variety of external environmental factors^1^. Ca^2+^ is a common second messenger in the signaling process of eukaryotic cells^2,3^. When plants are exposed to different extracellular stimuli, specific calcium signals are triggered and recognized by different calcium sensors, leading to the activation of downstream cascade systems^4^. Calcium sensors can be grouped into three main classes: calcium-dependent protein kinase (CDPK/CPK), calmodulin and calmodulin-like proteins (CaM and CaML), and calcium phosphatase-like subunit B proteins (CBL)^5^. Of these, CDPK is a class of serine/threonine protein kinases unique to plants and some native organisms, and due to their specific structure, CDPKs do not require the involvement of calmodulin and can directly sense and respond to calcium signals^6–8^.

Calcium-dependent protein kinases (CDPKs) are enzymes that directly use ATP-activated protein substrates and Ca^2+^ as a signal, mainly found in plants and protozoa^6,9^. A typical CDPK molecule consists of a polypeptide chain with four functional regions or structural domains, which are the variable N-terminal domain (VNTD), the Ser/Thr protein kinase domain (PKD), the junction domain (JD), and the C-terminal calmodulin-like regulatory structure with an EF chiral structure^10^. CDPK is widely distributed in plants and plays various roles in carbon/nitrogen metabolism, cytoskeleton regulation, stomatal movement regulation, growth regulation^11^, and plant resistance to biotic and abiotic stresses^12,13^. As an ancient gene family, CDPK genes are found in protozoa such as *Toxoplasma gondii* ^14^ and early lower plants such as green algae^15^, making them ideal for studying plant genetic evolution.

Numerous studies from different plant species have confirmed that CDPK genes play an important role in regulating plant growth and responses to various stimuli, including light, hormones, injury, abiotic and biotic stresses^15^. For instance, overexpression of MDCPK1A in tobacco can remove ROS accumulation and regulate the expression of stress-related genes, thereby significantly increasing cold and salt resistance^16^. In Brazilian rubber, three CDPK genes (CDPK1, CDPK3, and CDPK4) were isolated under low-temperature stress^17;^ the differential expression trend of CDPK1 indicated that these genes might play an essential role in the low-temperature stress tolerance of rubber^18^. Wang et al.^19^ identified 29 CDPK genes in tomato and mined the SiCDPK protein with STK-CAMK type protein kinase structural domain and a unique EF-hand type Ca protein-binding domain. Based on the eight CDPK genes cloned in Cavendish banana, MaCDPK3 was found to be a biomarker associated with drought cold and salt stress^20^. In grapes, 19 CDPK genes were identified, and the gene expression analysis in wild grapes showed high adaptive capacity and high-expression resistance to known adverse environmental conditions^21^. In strawberries, nine new CDPK genes were identified, and FaCDPK4 and FaCDPK11 genes were found to be involved in the abiotic stress response through interactions with other proteins, such as proteins involved in the ABA-dependent response to plant stress via Ca2+ signaling, especially RBOHs^22^. Although CDPK gene families in various crops have been increasingly studied^15^, systematic analysis of the CDPK gene family in white clover has not been comprehensive.

White clover (*Trifolium repens*), also known as white cheatgrass, dutch buckwheat, and creeping clover, is a perennial herb of the genus Trifolium in the Leguminosae family^23^. It is an allotetraploid forage legume used as a forage grass, now widely grown in temperate and subtropical regions^24^. It has environmental benefits in terms of biological nitrogen fixation and has medicinal properties, including the ability to clear heat, cool the blood, and calm the heart^25^. The suitable growth temperature for white clover is 19–24°C, when the low temperature comes in winter, it is easy to suffer from cold and wilts and withers earlier than other grass species such as early grassland and tall fescue; white clover turns green late and does not turn green evenly^26^. Therefore, improving the cold tolerance of white clover has become an important issue. However, the genetic knowledge of white clover as a non-model plant is very poor^27,28^. Until 2019, the genome sequence of white clover was published, and the issue about the genetic background of white clover was resolved^29^. Related researchers were able to study the function of genes at the genome level, which significantly contributed to the genetic improvement of white clover. For example, Ma et al. identified 133 R2R3-MYBs in the genome of white clover and characterized that some TrMYB genes could positively regulate anthocyanin accumulation and low-temperature response in white clover^30^.

Herein, we identified CDPK genes in white clover at the genome-wide level using the bioinformatics methods and systematically described their structural composition, chromosomal distribution, and genetic regulatory network by integrating various datasets. In addition, RNA-seq was used to investigate the response of CDPK genes to cold stress, and their expression profiles were confirmed by qRT-PCR. These findings will provide a reference for exploring the biological functions of CDPK in response to cold stress and its molecular mechanisms in white clover.

## Materials and methods

### Identification and classification of the TrCDPK genes in white clover

Genomic resource information for white clover was obtained from previous studies published, all files were provided by Stig Uggerhøj Andersen, Aarhus University^29^. DNA, CDS, and protein sequences were extracted from the white clover genome. The protein sequence of Arabidopsis CDPK sequences was obtained from the TAIR database. To identify the CDPK family members in white clover, the Arabidopsis CDPK sequences were used as the query to perform a BLASTP search of the genome of white clover^31^, with an evaluation setting of 1E–05, and the coverages were set as 80%. The HMM file including EF-hand (Pfam ID: PF13499) and serine/threonine protein kinase (Pfam ID: PF00069) was downloaded from the Pfam database, and the HMMER (evalue: 0.01) was performed to recognize and confirm the protein domains that were characterized as candidate CDPK proteins^32^. The annotation information of all candidate CDPK genes, such as genomic location, protein length, and intron number, was obtained from the white clover genome. Finally, all members of white clover CDPK genes were classified into groups based on similar CDPK genes in Arabidopsis.

### Phylogenetic analysis of the TrCDPK genes in white clover

Candidate white clover CDPK protein sequences were multiply aligned using MUSCLE with default parameters^33^. A phylogenetic tree was generated using the Neighbor-Joining method with 1 000 rapid bootstrap repeats using MEGA11 and protein sequences from Arabidopsis^34^. The TrCDPK genes were classified into different groups and subgroups based on the phylogenetic tree of AtCDPK and TrCDPK sequences.

### Motif composition distribution analysis of TrCDPK genes in white clover

The MEME program was used to identify the conserved motifs in white clover CDPK protein sequences^35^. MEME searching was performed across TrCDPK protein sequencing using the following parameters: (1) any number of repetitions of a single motif; (2) the maximum number of different motifs up to 10 motifs; (3) the minimum motif width with six amino acids, the maximum motif width of a motif with 50 amino acids. All results were displayed with TBtools^36^.

### Chromosomal location and gene duplication of CDPK genes in white clover

The information of the CDPK gene family chromosome physical location was extracted from the white clover genome annotation file, and the gene duplications were identified and characterized based on this information by software MCSanX with default parameter^37^. The chromosomal locations of CDPK genes in white clover were drawn with CIRCOS software^38^, while revealed replication between CDPK genes in white clover. The definition of gene duplication is as follows: (1) the alignment length covered >80% of the longer gene; (2) the identify of alignment region is greater than 80%; (3) only one duplication event gene was counted for tightly linked genes. According to the chromosomal location of CDPK genes, tandem duplication and segment duplication can be identified.

### Gene regulation network analysis of white clover CDPK genes

The gene regulatory network (GRN) information of Arabidopsis was obtained from the AraNet database (V2)^39^, including 22,894 Arabidopsis genes and 895,000 interactions (links). All proteins from white clover and Arabidopsis were searched twice against each other in BLAST, with e values cut off at 1e-05, and the highest scoring hit was identified as a homolog of the white clover gene or a homolog of the Arabidopsis gene. These two genes were identified as homologous pairs with two BLAST results. Then, the GRN of Arabidopsis was used to construct the GRN of white clover based on the information of the homologous pairs. Sub-networks containing the white clover TrCDPK gene were searched and analyzed, and the results were visualized using Cytoscape software^40^. Gene ontology (GO) enrichment analysis was performed on the sub-networks using topGO with the threshold level set to 0.05, showing the representation of the most important terms and designating the highly enriched terms as GRN functions, as described in the software protocol^41^.

### Expression analysis of white clover TrCDPK genes in response to cold stress

RNA-seq data have been reported that include eight time points in response to cold stress, which has been described in detail in our previous work and can be evaluated using accession number: PRJNA781064. The Salmon software (version 0.12.0) was used to map the RNA-seq reads to the transcript sequences of white clover genomes, and the expression levels (FPKM values) of each gene were calculated by a subroutine of Salmon^42^. These expressional data were transformed using the “log2” function and centered using the “scale” function of the R program. Then, all expression data were clustered and plotted using the “heatmap.2” function of the ggplots package. Meanwhile, the TrCDPK gene expression data were extracted and listed in Supplementary Materials.

### Plant growth and Qrt-PCR analysis

Seeds of white clover cv. Haifa were purchased from Barenbrug China Ltd. Com. (Beijing, China). The seeds were germinated and planted in pots of about 10–15 plants each containing perlite and vermiculite (in a 3:1 ratio), as we described previously^43^. The growth conditions were a light cycle of 14 h at 24°C and a dark cycle of 10 h at 18°C and were irrigated once a day with a half-strength Hoagland solution. After four weeks of growth, they were randomly assigned into four groups for cold stress treatment. We sampled the following four nodes (0 min (control), 30 min, 1 h, and 3 h, respectively) after treatment, working at 4°C. For each group, three samples were randomly selected from the seedlings and pooled into one biological replicate. All samples were frozen in liquid nitrogen and then stored in a −80°C refrigerator. Total RNA was extracted from white clover seedlings at different time points of cold stress (0 min (control), 30 min, 1 h, and 3 h, respectively) at 4°C using the Total Plant RNA Extraction Kit (Tiangen, Beijing, China). cDNA was reverse transcribed using the PrimeScript RT kit (Toyobo, Shanghai, China). Primers were designed using Primer3 (Table S1)^44^. qRT-PCR was performed using a Light Cycler® 96 system (Roche, Rotkreuz, Switzerland) and SYBR Premix Ex TaqTM II (Toyobo, Shanghai, China). The program for qRT-PCR was 95°C for 2 minutes, 40 cycles, 95°C for 30 seconds, 55°C for 30 seconds, and 72°C for 1 minute. Three replicates were performed for each experiment. The relative expression of the CDPK gene was calculated using 2(-∆∆Ct) analysis^45^.

## Results and analysis

### Identification of CDPK genes in white clover

To identify the CDPK gene family members in white clover, the CDPK protein sequence of Arabidopsis thaliana was used as the reference sequence and then searched in the genome-wide database of white clover. Subsequently, the candidate genes were subjected to protein structure analysis to determine their Protein kinase and EF-hand structural domains. A total of 50 TrCDPK genes were successfully identified from the white clover genome by multiple sequence alignment, based on the amino acid sequence of the white clover CDPK gene family (Table 1). As shown in Table 1, the genomic information of the TrCDPK genes, including name, locus, chromosomal locations, group, intron, and protein length (aa), was retrieved and summarized. Among these 50 TrCDPK genes, TrCDPK50 had the longest protein length with 1053 amino acid residues, while TrCDPK40 had the shortest amino acid length with 254 amino acid residues. In addition, the distribution of intron in these TrCDPK genes ranged from 4 to 24. Most of the TrCDPK genes contained 6 or 7 introns. TrCDPK50 had the highest number of intron (24). The white clover had 16 chromosomes and these TrCDPK genes were unevenly distributed on the chromosomes of the white clover.

**Table 1.** Summary of TrCDPK genes identified in the white clover.

Name	Locus	Chromosomal locations	Group	Intron	Length(aa)
TrCDPK01	chr1.jg4121	Tr1O:29342857–29350032	I	9	652
TrCDPK02	chr11.jg3046	Tr3P:19475967–19479305	I	6	490
TrCDPK03	chr11.jg7419	Tr3P:49699451–49702794	I	6	490
TrCDPK04	chr12.jg3340	Tr4P:22292097–22296685	I	6	525
TrCDPK05	chr13.jg1707	Tr5P:11919089–11925479	I	6	484
TrCDPK06	chr15.jg4289	Tr7P:28188470–28195896	I	6	583
TrCDPK07	chr16.jg1647	Tr8P:11857550–11861634	I	6	501
TrCDPK08	chr4.jg11768	Tr4O:82749599–82756663	I	8	543
TrCDPK09	chr6.jg2442	Tr6O:16667798–16670593	I	6	496
TrCDPK10	chr6.jg2448	Tr6O:16751970–16754761	I	6	496
TrCDPK11	chr7.jg7026	Tr7O:43824351–43827109	I	6	567
TrCDPK12	chr8.jg330	Tr8O:2723857–2728472	I	6	524
TrCDPK13	chr8.jg5721	Tr8O:40260485–40264967	I	6	577
TrCDPK14	chr9.jg3491	Tr1P:23497749–23502201	I	6	577
TrCDPK15	chr9.jg4545	Tr1P:30742384–30748425	I	6	606
TrCDPK16	chr9.jg4549	Tr1P:30758875–30767018	I	7	604
TrCDPK17	chr9.jg4589	Tr1P:31051402–31054840	I	7	606
TrCDPK18	chr10.jg1626	Tr2P:11278235–11282359	II	7	536
TrCDPK19	chr11.jg7540	Tr3P:50482871–50486987	II	7	538
TrCDPK20	chr12.jg6833	Tr4P:44711922–44716107	II	7	511
TrCDPK21	chr13.jg2122	Tr5P:14691325–14695563	II	7	515
TrCDPK22	chr13.jg282	Tr5P:1691449–1695649	II	7	454
TrCDPK23	chr13.jg6349	Tr5P:41734498–41736909	II	7	519
TrCDPK24	chr16.jg6213	Tr8P:44320503–44324769	II	7	413
TrCDPK25	chr16.jg6322	Tr8P:44999858–45004251	II	7	557
TrCDPK26	chr3.jg10857	Tr3O:71946757–71950273	II	7	525
TrCDPK27	chr4.jg4021	Tr4O:29365007–29368523	II	7	525
TrCDPK28	chr4.jg4266	Tr4O:31247499–31249871	II	7	519
TrCDPK29	chr5.jg6878	Tr5O:46579663–46583375	II	7	529
TrCDPK30	chr5.jg6984	Tr5O:47190935–47194811	II	7	529
TrCDPK31	chr5.jg6985	Tr5O:47195769–47197955	II	4	410
TrCDPK32	chr7.jg2061	Tr7O:12628766–12633112	II	7	574
TrCDPK33	chr1.jg1928	Tr1O:14027305–14040793	III	22	1013
TrCDPK34	chr1.jg3552	Tr1O:25810144–25817921	III	8	555
TrCDPK35	chr1.jg3553	Tr1O:25820401–25824560	III	7	528
TrCDPK36	chr11.jg8874	Tr3P:59581528–59592044	III	8	510
TrCDPK37	chr11.jg8876	Tr3P:59596963–59603478	III	10	576
TrCDPK38	chr11.jg8877	Tr3P:59611160–59615352	III	8	519
TrCDPK39	chr12.jg4640	Tr4P:30428711–30431256	III	7	532
TrCDPK40	chr15.jg2533	Tr7P:16933744–16938277	III	7	254
TrCDPK41	chr16.jg464	Tr8P:3255395–3259046	III	6	479
TrCDPK42	chr8.jg5032	Tr8O:35936316–35940504	III	7	490
TrCDPK43	chr9.jg2547	Tr1P:17241045–17244635	III	7	535
TrCDPK44	chr9.jg2588	Tr1P:17548790–17554644	III	6	531
TrCDPK45	chr9.jg6904	Tr1P:46606550–46612048	III	6	531
TrCDPK46	chr11.jg6545	Tr3P:43486048–43493182	IV	10	590
TrCDPK47	chr2.jg5591	Tr2O:37510045–37516429	IV	10	589
TrCDPK48	chr5.jg1099	Tr5O:7718463–7722608	IV	11	554
TrCDPK49	chr5.jg5438	Tr5O:36322713–36329697	IV	10	588
TrCDPK50	chr5.jg999	Tr5O:6993561–7004058	IV	24	1053

### Phylogenetic analysis of CDPK genes in white clover

**Figure 1.**
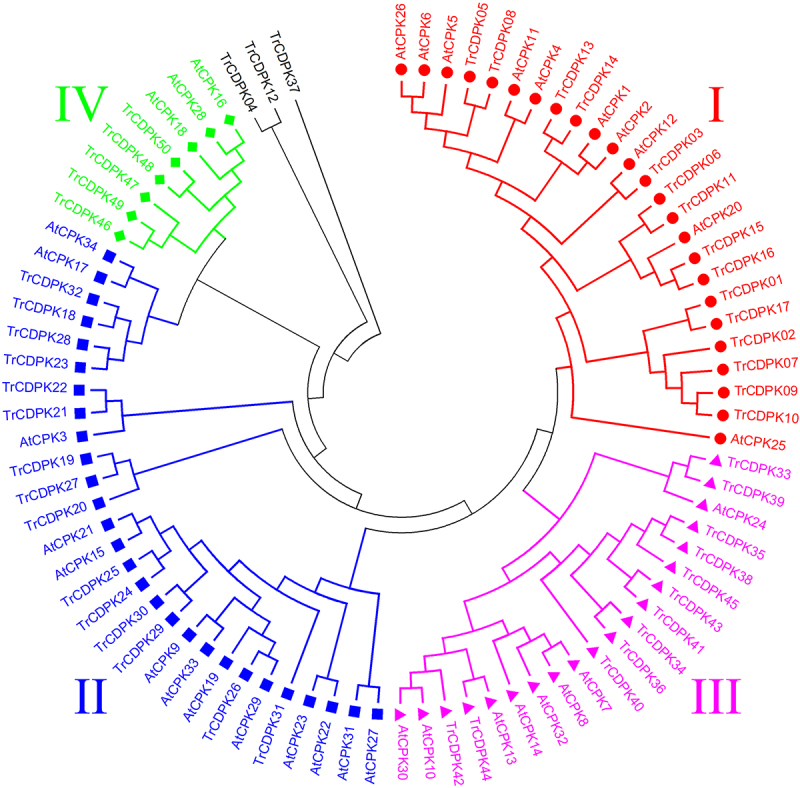
Phylogenetic analysis of white clover CDPK proteins.

To further explore the phylogenetic relationship of the CDPK genes in the white clover, an unrooted phylogenetic tree was reconstructed with 50 TrCDPKs using neighbor-joining methods (Figue 1). This unrooted tree intuitively reflected the evolutionary status and grouping attribution of 50 members of the CDPK gene family. Phylogenetic analysis of CDPK genes in Arabidopsis thaliana and white clover revealed that these genes were clustered into four branches, named Group I–Ⅳ, corresponding to the results obtained through structural similarity, respectively. Further analysis of the clustering results of CDPK members revealed that the gene structures in the homologous gene pair were similar. As shown in Figure 1, the number of Group II proteins was the largest, including 15 white clover and 13 Arabidopsis CDPK proteins, respectively. The first group included 15 CDPK proteins from white clover and 10 CDPK proteins from Arabidopsis. The third group included 12 CDPK proteins from white clover and eight CDPK proteins from Arabidopsis. The fourth group had the least number, including only five CDPK proteins from white clover and three CDPK proteins from Arabidopsis, indicating that it is far from each other. There were three members in the phylogenetic tree that were not grouped, TrCDPK4, TrCDPK12, and TrCDPK37, respectively. At the same time, the molecular phylogenetic tree showed that the distribution of CDPK genes in white clover and Arabidopsis thaliana was highly consistent.

The NJ tree was constructed from the amino acid sequences of TrCDPK using MEGA4 with 1000 bootstrap replicates. The white clover CDPK proteins were grouped into three groups (Group I labeled as red solid circles, Group II labeled as blue solid squares, Group III labeled as pink solid triangles, and Group IV labeled as green solid diamonds).

### Motif composition distribution analysis of TrCDPK proteins in white clover

**Figure 2.**
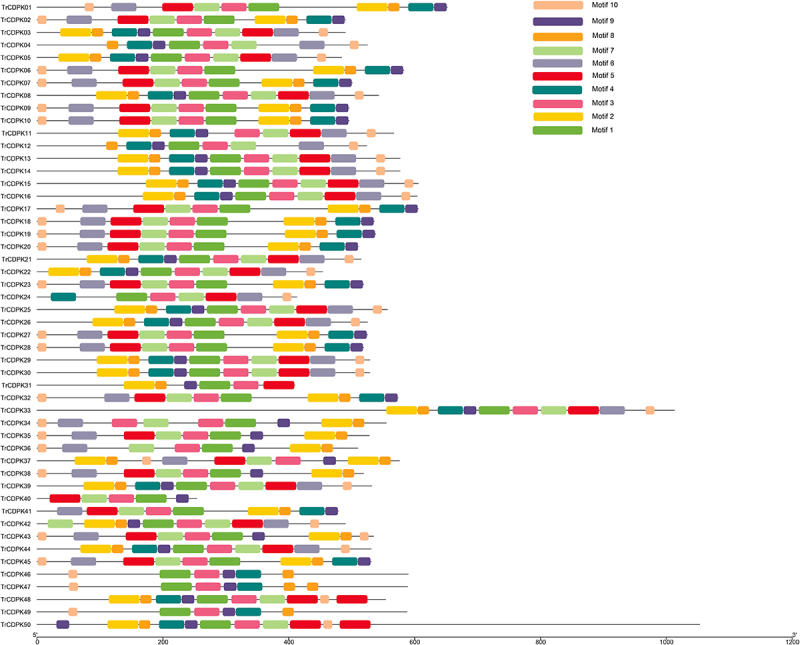
Distribution of conserved motifs of TrCDPK genes in white clover.

To investigate the protein structural and functional diversity of TrCDPKs, motif analyses were performed based on the phylogenetic relationship (Table 1 and Figure 1). Generally, 10 conserved motifs within the white clover CDPKs were identified using online MEME tools, which can help us investigate their function (Figure 2 and Figure S1). As mentioned above, the unrooted phylogenetic tree showed that TrCDPKs were broadly divided into four major subgroups. The results showed that they have similar gene structure distribution patterns in the same subfamily. Most of these TrCDPKs in Group I contained 10 motifs in total, while TrCDPK04, and TrCDPK12 contained the same conserved motifs without Motif 2, and Motif 5. Meanwhile, the conserved gene structures revealed similar motifs among groups. Three of the motifs (motif 1, 3, and 7) were shared by all the CDPK proteins. The motif analysis results illustrated that conserved motif structures within each group supported their close evolutionary relationship, while there might be functional divergences between different groups.

### Chromosome localization and gene duplication analysis of TrCDPK genes in white clover

**Figure 3.**
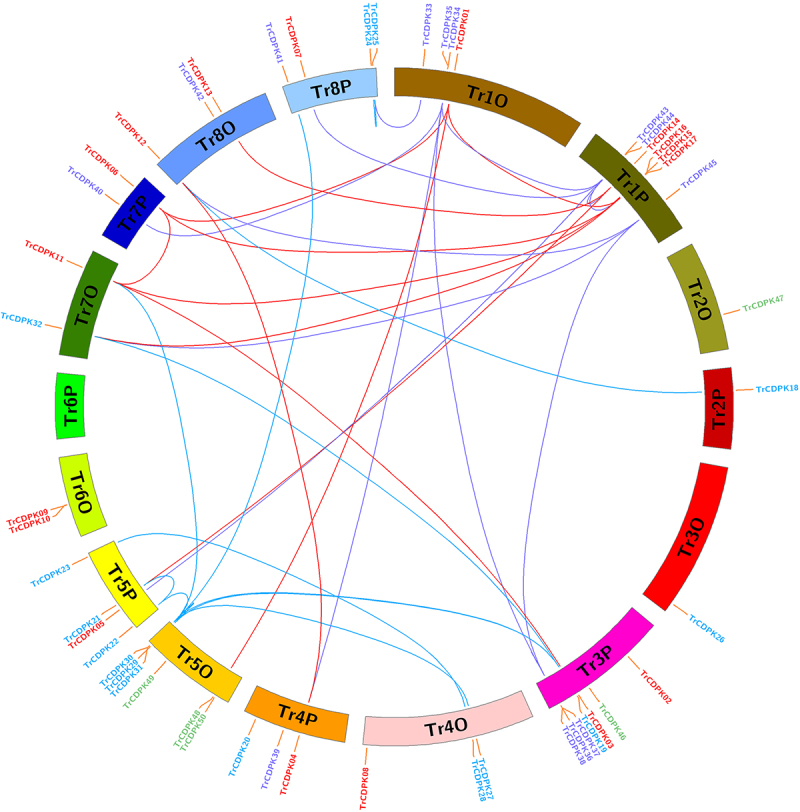
Chromosome distribution and expansion analysis of CDPK genes in white clover.

To determine the evolution and expansion of CDPK genes, we used MCScanX and Circos software to construct the distribution of CDPK genes across chromosomes (Figure 3). As shown in Figure 3, 50 TrCDPK genes were distributed across 16 chromosomes, mainly chromosome TrChr1O, TrChr1P, TrChr3P, TrChr5O, TrChr5P, TrChr8O, and TrChr8P, respectively. The fewest number of genes were found in TrChr2O, TrChr2P, and TrChr3O with only one CDPK genes. Using sequence alignment, mainly through gene duplication, 34 out of 50 genes were duplicated and divided into two categories. There were 34 segment duplications (SD) caused by the amplification of CDPK genes on different chromosomes and 4 tandem duplications (TD) resulting from the generation of CDPK gene clusters. The SD and TD genes were mainly found in TrChr1O, TrChr1P, TrChr3P, TrChr5O, and TrChr5P while other chromosomes only contained SD genes. The results have suggested chromosome doubling helps to bring about CDPK expansion in white clover, and distributions of TrCDPK were similar between some doubling chromosomes, for example, chromosomes TrChr8O and TrChr8P, chromosome TrChr5O and TrChr5P. The regions containing TD genes were hot regions of gene distribution. These duplications may have led to the expansion of the TrCDPK gene family in the white clover genome.

Red lines show duplications between members of Group I, blue lines show duplications between members of Group II, purple lines show duplications between members of Group III, and green lines show duplications between members of Group IV.

### Genetic regulation network analysis of white clover CDPK genes

**Figure 4.**
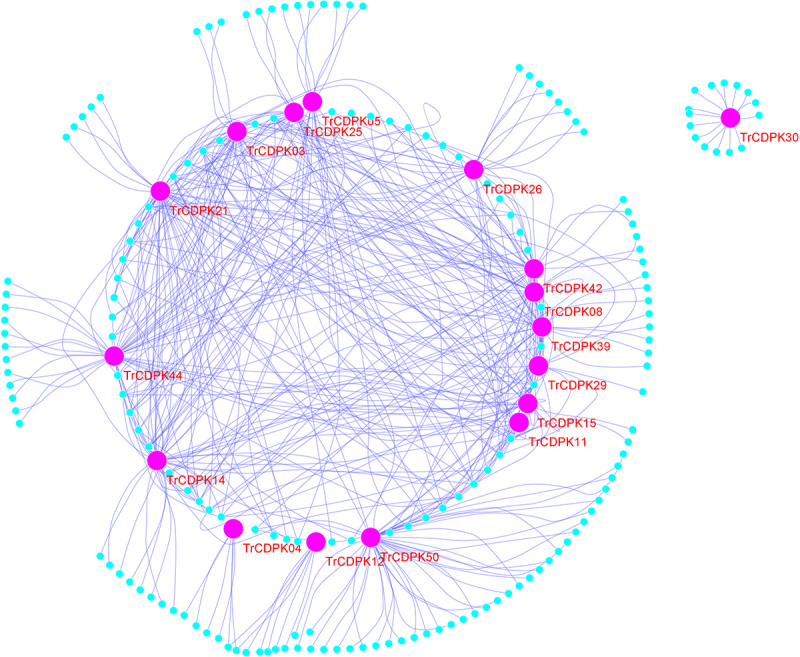
Gene regulatory network analysis of TrCDPK genes and their interactions in white clover.

**Figure 5.**
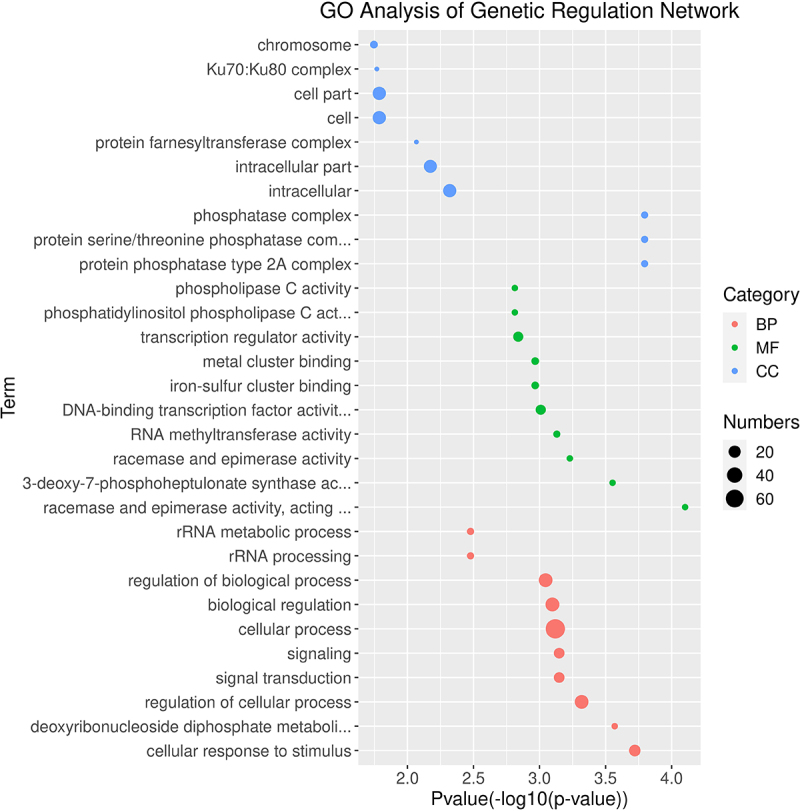
Gene Ontology enrichment analysis of interaction genes with TrCDPK genes.

Gene regulation networks (GRN) can be an effective method for studying gene function, and we have predicted GRNs of TrCDPK and their interacting genes according to the public interaction database. As shown in Figure 4, the GRNs have consisted of 195 genes and 414 interactions. From GRNs, we found that most of TrCDPK interacts with dozens of functional genes, which is consistent with the function of TrCDPK in the regulation of transcription. For example, TrCDPK03 interacted with 31 genes, TrCDPK21 with 39 genes, TrCDPK39 with 26 genes, and TrCDPK50 with 46 genes; the results indicated these TrCDPK genes played important roles in white clover lifespan. Gene Ontology (GO) annotation of these interacting genes was searched, and the topGO package on the R platform was used for GO enrichment analysis. The results showed that they were mainly distributed in the cell part, see Figure 5, which supported TrCDPK genes also functioned in the intracellular part. In terms of molecular function, the CDPK gene acted mainly in terms of phosphatase C activity and transcriptional regulator activity. This result suggested that CDPK genes can play an important role in intracellular phosphorylation pathways. In biological processes, CDPK genes can act in signal transduction, biological regulation, and cellular response to stimulus, which are plant popular descriptions in response to abiotic stress. These results could indicate that CDPK genes may play an active role in response to abiotic stresses.

Gene regulatory network (GRN) of TrCDPK genes and their interactions were generated based Arabidopsis interactions, which was displayed with Cytoscape. Pink nodes correspond TrCDPK genes, while steelblue nodes correspond the genes interacted with TrCDPK genes, the violet lines represented interactions in white clover.

The GO enrichment analysis showed the involvement of interaction genes with TrCDPK genes in biological processes, molecular functions, and cellular components. Red dots represent GO terms from biological process (BP), green dots represent GO terms from molecular function (MF), while blue dots represent GO terms from cellular component (CC). Dot size represents the number of genes involving in the GO term, the X-axis is the p-value of topGO enrichment analysis, with -log10 transformation, -log10 (p), while the Y-axis is GO terms.

### Expression analysis of TrCDPK genes in response to the cold stress

**Figure 6.**
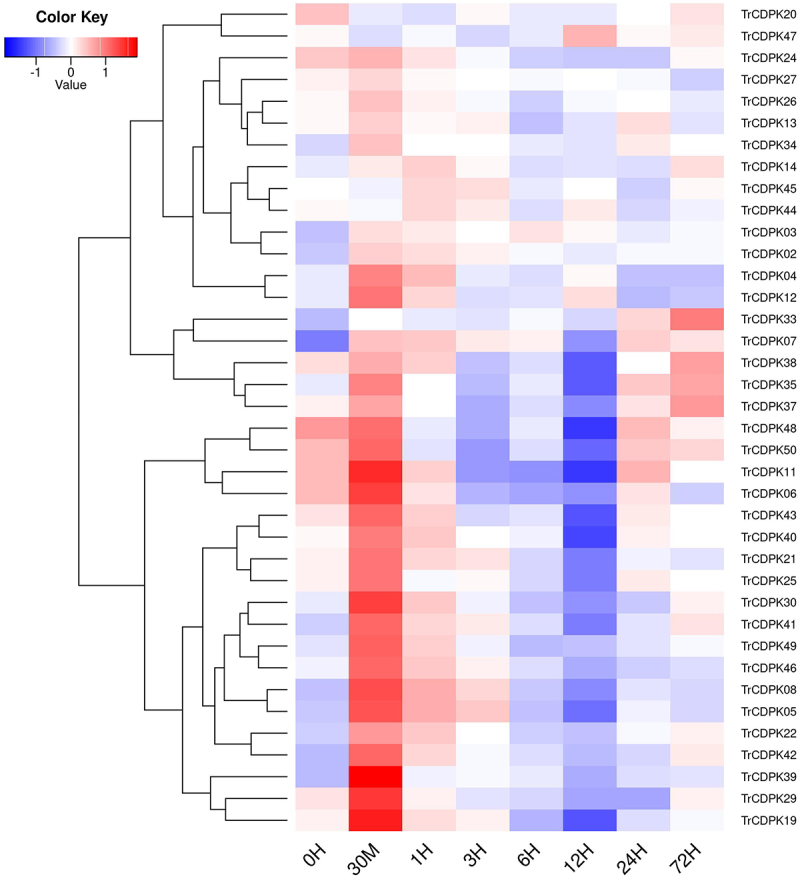
The expressional profiles of TrCDPK genes in response to cold stress.

To investigate the function of the TrCDPK genes in response to abiotic stress, we used previous RNA-seq data under cold stress to evaluate their expression profile. Cold stress treatment of white clover was performed at 4°C and RNA-seq analysis was performed at eight time points including 0 H, 30 M, 1 H, 3 H, 6 H, 12 H, 24 H, and 72 H. All expressed TrCDPK genes (FPKM values >1) were collected and their expression values (FPKM) were grouped using violin plots, which showed that TrCDPK genes increased in response to cold stress (Figure S2). In particular, TrCDPK genes were rapidly activated within 30 min and maintained high expression levels in subsequent stages, the results suggested that TrCDPK genes play a key role in the early stage of cold stress. Through clustering of the expression profiles of these TrCDPK genes and displaying them using heat map functions, the result showed that most TrCDPK genes were highly expressed within 30 min in response to cold stress (Figure 6). This finding coincided with the violin plot analysis and confirmed their rapid response to cold stress. Some of these TrCDPK members were clearly highly expressed within 30 min, such as TrCDPK08, 19, 21, 29, 30, 39, 41, 42, 49. Combined with their pivotal functions in previous GRNs, such as TrCDPK21 and TrCDPK39, they would be assigned to rapid and critical regulatory roles in response to cold stress.

The expressional profiles of TrCDPK genes were retrieved from RNA-seq database with accession numbers: PRJNA781064. There were eight time points, including 0 H, 30 M, 1 H, 3 H, 6 H, 12 H, 24 H, and 72 H, each time point has three biological replications. Mean expression levels (FPKM values) were measured by Salmon software, and they were displayed using ggplots package of R platform.

### Qrt-PCR validation of TrCDPK genes expression in response to cold stress

**Figure 7.**
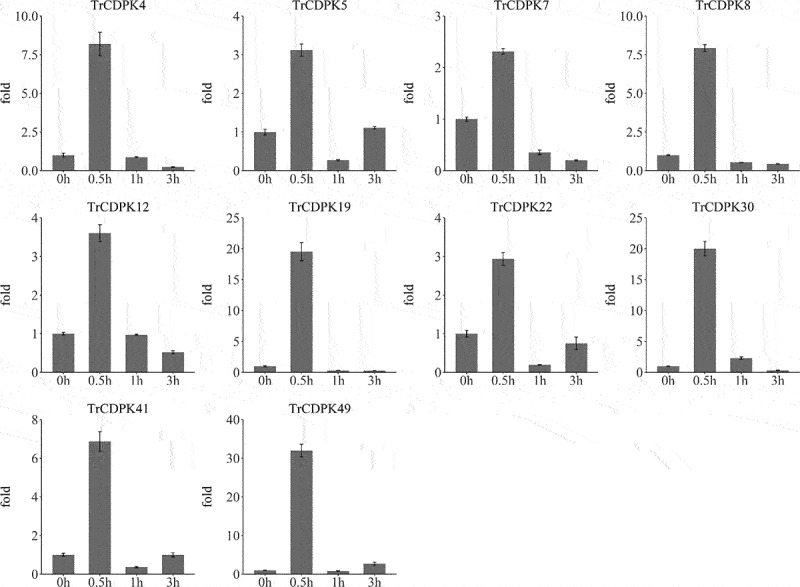
Qrt-PCR analysis of TrCDPK genes in response to cold stress.

To examine the rapid response to cold stress, we performed qRT-PCR analysis of 10 TrCDPK genes at four time points, consisting of 0 h, 30 min (0.5 h), 1 h, and 3 h. The results of qRT-RCR analysis confirmed their rapid and high expression in response to cold stress (Figure 7). The expression of the CDPK gene family in white clover changed to some extent under cold stress. The expression of TrCDPK4, TrCDPK8, TrCDPK19, TrCDPK30, and TrCDPK49 changed significantly under cold stress. The expression of all these genes was highest at 0.5 h of cold stress, with the highest expression of TrCDPK49 being approximately 32.0 times higher than that of the control (0 h). All TrCDPK genes showed a sharp increase followed by a slight decrease. qRT-PCR and RNA-seq results were consistent, revealing a similar expression pattern of TrCDPK genes in response to cold stress. These findings indicated that the TrCDPK genes actively participated in the early response to cold stress and played a key role in regulating gene expression in white clover.

The X-axis represents time points in response to cold stress, and the Y-axis represents relative expression level of TrCDPK genes, which sets the expression level at “0 h” time point as 1. The expression level were calculated using the 2^−ΔΔCT^ method as method section described.

## Discussion

Cold stress affected the normal growth and development of white clover and did not allow normal overwintering and re-growth^46^. Studying the response mechanism of white clover to cold stress is an important way to improve the yield and quality of white clover. Plant CDPK genes and their signaling cascades can regulate plant growth and development and are involved in hormone signaling, abiotic stress signaling, and innate immune responses^47,48^.

Members of the CDPK gene family have now been identified and extensively studied in numerous plants^49^. CDPK genes have been identified in many species by whole-genome sequencing and in large numbers. There are 34 CDPK genes in the Arabidopsis genome^6^, 31 CDPK genes in the rice genome^50^, and 41 CDPK genes in diploid cotton^51^. In horticultural crops, CDPK has also been identified in several species, such as 29 CDPK genes in the tomato genome^52^ and 19 CDPK genes in both cucumber and grape^21,53^. During evolution, the CDPK gene family has shown a high degree of structural conservation from mosses to angiosperms^49^. The gene family is generally divided into four subfamilies with varying degrees of divergent amplification, of which subfamily IV has the smallest number of genes and is also the oldest^49^. In this study, 50 TrCDPK gene family members were identified from white clover using the bioinformatic methods. White clover contains mainly 16 chromosomes and has a genome size of approximately 841 Mb^29^. This suggests that there is no linear relationship between the number of CDPK gene family members and the genome size of the species, and it is speculated that some TrCDPK genes lost their function and gradually evolved and died out or evolved into other genes during the evolution of white clover due to the pressure of natural selection.

Increasing reports are showing that CDPK genes play an important role in signal transduction, and plant response to environmental stimuli, especially in response to biotic or abiotic stresses^6,54^. In the present study, we reconstructed 414 gene regulatory networks based on public interaction databases. A large proportion of TrCDPK genes have many interacting genes; for example, TrCDPK03 interacts with 31 genes, TrCDPK21 interacts with 39 genes, TrCDPK39 interacts with 26 genes, TrCDPK50 interacts with 46 genes, and so on. Based on the gene regulatory network and gene function annotation analysis, we found that TrCDPK50 regulated the chr12.jg1202 gene and both were enriched in the signal transduction pathway in biological processes. The chr12.jg1202 gene was a CBL-interacting protein kinase 2-like protein (CIPK). Previous studies have found that the CBL-CIPK complex in Arabidopsis can mediate plant responses to various external stresses, including drought, cold, and wounding^55,56^. This could suggest that the TrCDPK genes in white clover may respond with the CBL-CIPK complexes to mediate plant responses to cold stress. According to the results of GO enrichment analysis, most of the genes are focused on processes such as signal transduction, cellular response to stimuli and biological regulation. These results also confirm that the function of TrCDPK genes is highly conserved in white clover. Similar insights have been reported in other plants; for example, CDPKs play an important role in abiotic stress tolerance through hormone signaling and reactive oxygen species (ROS) signaling in response to various environmental stresses such as cold, salt, or drought stress^57;^ in Arabidopsis, AtCPK10 interacts with the heat shock protein HSP1 and is involved in drought tolerance regulation through abscisic acid (ABA) and Ca^2+^-mediated stomatal regulation^54;^ Sheen et al. found that the expression of Arabidopsis AtCPK1 and AtCPK2 was significantly increased under drought and salt-induced conditions^58^, and Arabidopsis AtCPK10 and AtCPK30 were involved in signal transduction in response to ABA and abiotic stresses. These imply that CDPK genes play an important function in plant resistance to the environment, which is consistent with our findings.

It is well known that Ca^2+^ plays a huge role in plant growth and development as a ubiquitous second messenger in the plant signaling system. When plants are stimulated by the environment and growth and development, stimuli triggered by external factors, such as temperature, light, salinity, and osmotic pressure, produce different calcium ion changes. Specific calcium ion receptors can recognize and sense these changes and then play a role in regulating gene expression through a series of cascade responses^59^. Through research, CDPK genes sense changes in Ca^2+^ concentration through EF-hand structures, release self-inhibition, activate kinase structural domains, and then transmit information to regulate physiological changes in plants, which are widely involved in plant growth, development, and morphological construction^4^. In the present study, the expression of TrCDPK family members changed significantly after cold stress, especially in the early stages of cold stress, as shown in Figure S2. It showed a significant upregulation of TrCDPK gene expression after 0.5 h of cold stress. The expression of 32 TrCDPK genes was up-regulated at 0.5 h of cold stress, with TrCDPK4, TrCDPK8, TrCDPK19, TrCDPK30, and TrCDPK49 being the most significantly up-regulated. The results of qRT-PCR also confirmed the expression profile. These results demonstrate the ability of TrCDPK genes to respond rapidly to cold stress. This finding is consistent with other plants; for example, in Solanum habrochaites, Li et al. demonstrated that the expression of ShCDPK5, ShCDPK6, ShCDPK12, ShCDPK 15, ShCDPK26, ShCDPK29, and ShCDPK33 changed significantly under cold stress; after 0.5 h of cold stress, the expression of ShCDPK5 and ShCDPK6 was significantly up regulated^60;^ 12 MdCDPK genes were able to respond rapidly in *Medicago truncatula* under cold stress. These genes play an important role in the signaling pathway of CDPK genes in response to cold stress. Besides, OsCDPK13 and OsCPK17 are considered to be important signaling components in rice in response to cold stress^61,62;^ In tomato, CPK27 is a positive regulator of cold acclimation and responds to cold stress by participating in the regulation of ABA, ROS, NO, and MPK signaling pathways^63^. However, the specific molecular mechanism of TrCDPK in response to cold stress is unclear, and more experiments are needed to explore the function of CDPK genes.

## Conclusion

In the present study, 50 members of the CDPK gene family in white clover were identified and characterized. The following analysis was performed: gene identification and classification, phylogenetic and motif composition distribution analysis, chromosomal mapping and gene duplication analysis, genetic regulation network analysis and gene ontology annotation, gene expression analysis, and quantitative real-time reverse transcription PCR (qRT-PCR) analysis. The expression profiles of the TrCDPK genes in response to cold stress were assessed from RNA-seq data, which confirmed their regulatory functions in the cold stress response. Additionally, a white clover GRN was reconstructed using Arabidopsis interaction data. The qRT-PCR results directly revealed that the CDPK genes play a crucial role in response to early cold stress in white clover. The identification and systematic study of the CDPK genes in white clover will help to better explore the function of integrating the Ca^2+^ signaling pathway in white clover to adapt to cold stress.

## Supplementary Material

Supplemental Material
