# Supplementary material for: Genome-wide analysis of the CDPK gene family and their important roles response to cold stress in white clover
Source: Plant Signal Behav. 2023 May 18;18(1):2213924. doi: 10.1080/15592324.2023.2213924 (PMC10197994; doi:10.1080/15592324.2023.2213924)
Supplement: Supplemental Material [file KPSB_A_2213924_SM4421.zip › Table S1.docx]

**Table S1 Primers used for qRT-PCR analysis of the TrCDPK genes**

| **Name** | **Forward primer sequences (5’→3’)** | **Reverse primer sequences (5’→3’)** |
| --- | --- | --- |
| TrCDPK4 | TGAAGAGTTTACTGACCCTGT | ATCATTTGTTGTTTTTGGCGA |
| TrCDPK5 | ATACATGCCGTGGATCTTTG | TTTACGGCCAAGAGTGTAGA |
| TrCDPK7 | TTTTGGGCAGAAACTGAACC | AGAATTGCTTCAGACGTGAC |
| TrCDPK8 | CAGGTCAAGTTTTCACCGAT | TATGTCCCTTCAATACCGCA |
| TrCDPK12 | ATGAGAAGACTTGGAAGGGA | CGTGCATTAAACCTTTGTAGC |
| TrCDPK19 | CTTCTGGTCTTAGGAACAACC | ATGTTACTCCAAATTGACCTCT |
| TrCDPK22 | CAAAATGGGAAATCGCATCAG | CATCGGTTTGCCTAAGACAC |
| TrCDPK30 | AGCCAGAGAATTTCTTGTTGTC | AGGGACACCACTAAGTAAGAT |
| TrCDPK41 | TTACTATATGGCACCGGAGG | CACTGTCAGAAATCTGAGGC |
| TrCDPK49 | TGAAGGCCTTATCTGGACAT | CCTGAAGATGACAAAAGGCA |
| Action | TGCTTGATTCCGGTGATGGTGTG | TTCTCGGCAGAGGTACTGAAGGAG |
