# Supplementary figures and images for: Genome-wide analysis of the CDPK gene family and their important roles response to cold stress in white clover
Source: Plant Signal Behav. 2023 May 18;18(1):2213924. doi: 10.1080/15592324.2023.2213924 (PMC10197994; doi:10.1080/15592324.2023.2213924)

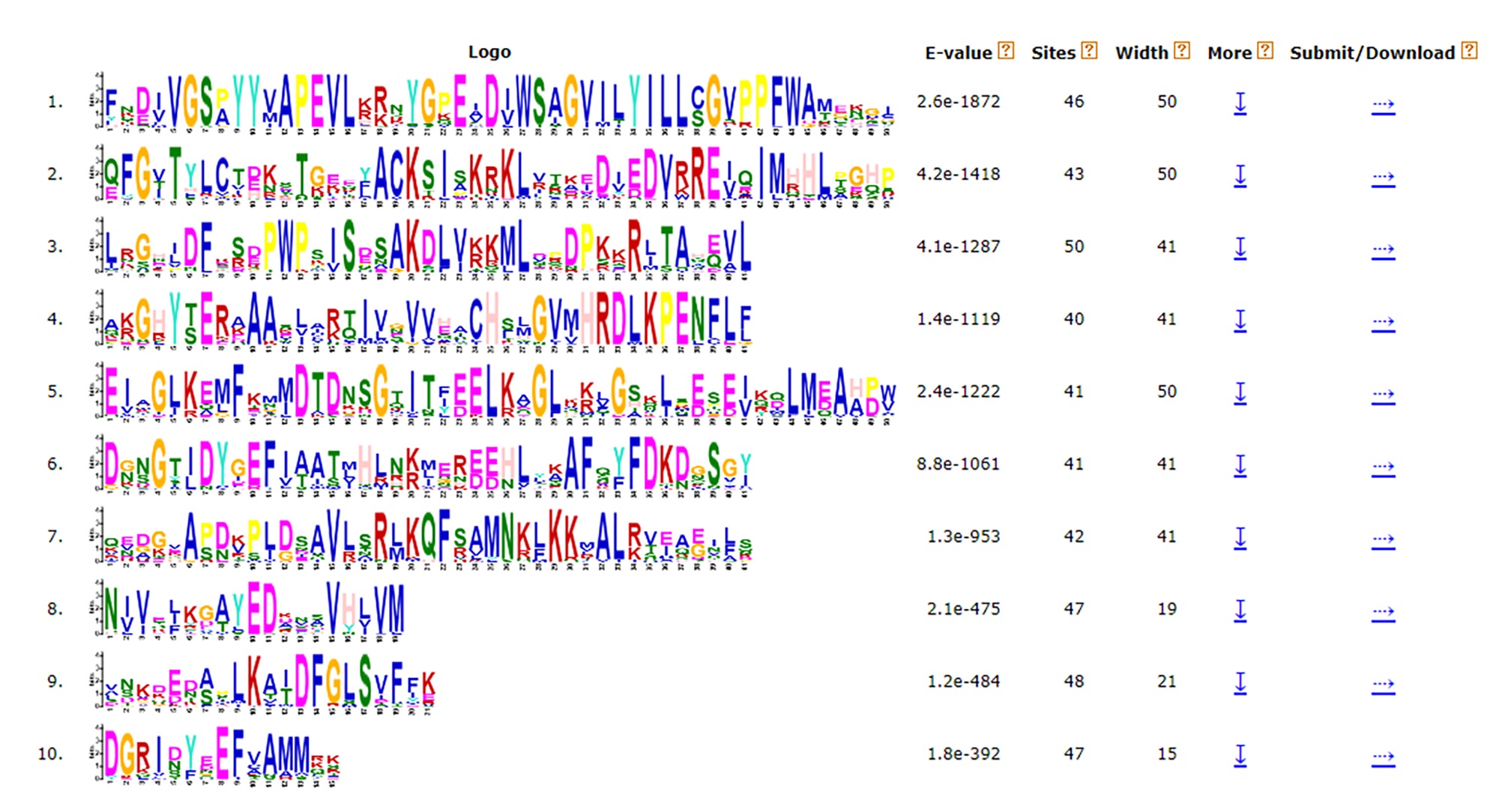

Supplement: Supplemental Material [file KPSB_A_2213924_SM4421.zip › Figure S1 Sequence logos for motifs identified from TrCDPK genes.jpg]

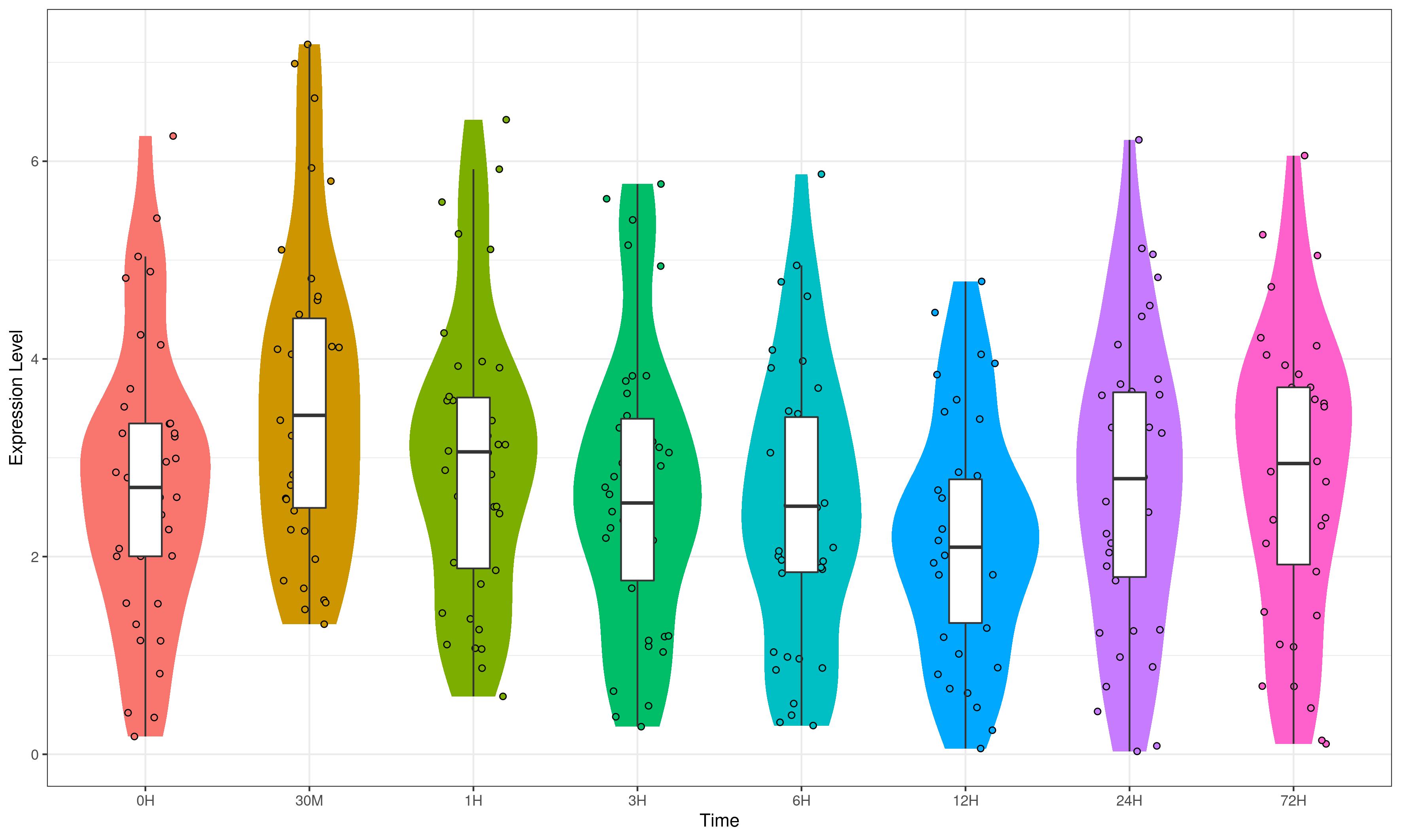

Supplement: Supplemental Material [file KPSB_A_2213924_SM4421.zip › Figure S2 The violin plot of all TrCDPK genes based their expression levels in response to cold stress..jpg]
